# Case Report: Two cases of adrenal anastomosing hemangioma misdiagnosed as angiosarcoma

**DOI:** 10.3389/fonc.2025.1534233

**Published:** 2025-05-21

**Authors:** Dong-Lin He, Run-Lin Feng, Xin Guo, Chang-Xing Ke

**Affiliations:** ^1^ Department of Urology, The Second Affiliated Hospital of Kunming Medical University, Kunming, China; ^2^ Department of Pathology, The Second Affiliated Hospital of Kunming Medical University, Kunming, China

**Keywords:** anastomosing hemangioma, adrenal gland, misdiagnosis, surgical resection, differential diagnosis

## Abstract

Anastomosing hemangioma (AH) is a benign vascular tumor that has gradually been recognized in recent years, initially identified in the genitourinary tract. AH located in the adrenal gland is extremely rare, and the atypical preoperative symptoms and imaging findings, as well as the postoperative pathological differential diagnosis, often lead to misdiagnosis. We herein report two case of adrenal AH that was misdiagnosed as adrenal angiosarcoma and review the relevant literature.

## Introduction

AH is a rare benign vascular tumor that primarily occurs in middle-aged and elderly individuals. It was first reported in 2009 and named anastomosing hemangioma of the genitourinary tract (1). The kidney is the most commonly involved organ in AH, followed by soft tissue and bone, especially the paravertebral region (2). Attributable to the lack of specific imaging features, preoperative differential diagnosis is very difficult. Even postoperative pathological diagnosis can be challenging due to insufficient awareness of AH. Owing to the interconnected vascular lumina within the tumor, lined by hobnail endothelial cells with mild atypia, differentiation from well-differentiated angiosarcoma is often required. Although long-term follow-up studies have shown that adrenal AH is a benign or indolent vascular tumor, the unclear preoperative diagnosis leads to surgical resection remaining the main treatment for this tumor (3). Here, we report two cases of adrenal AH that were misdiagnosed as adrenal angiosarcoma to enhance the awareness of this rare disease among pathologists and promote accurate diagnosis and precise treatment in clinical practice.

## Case report 1

**Figure 1:**
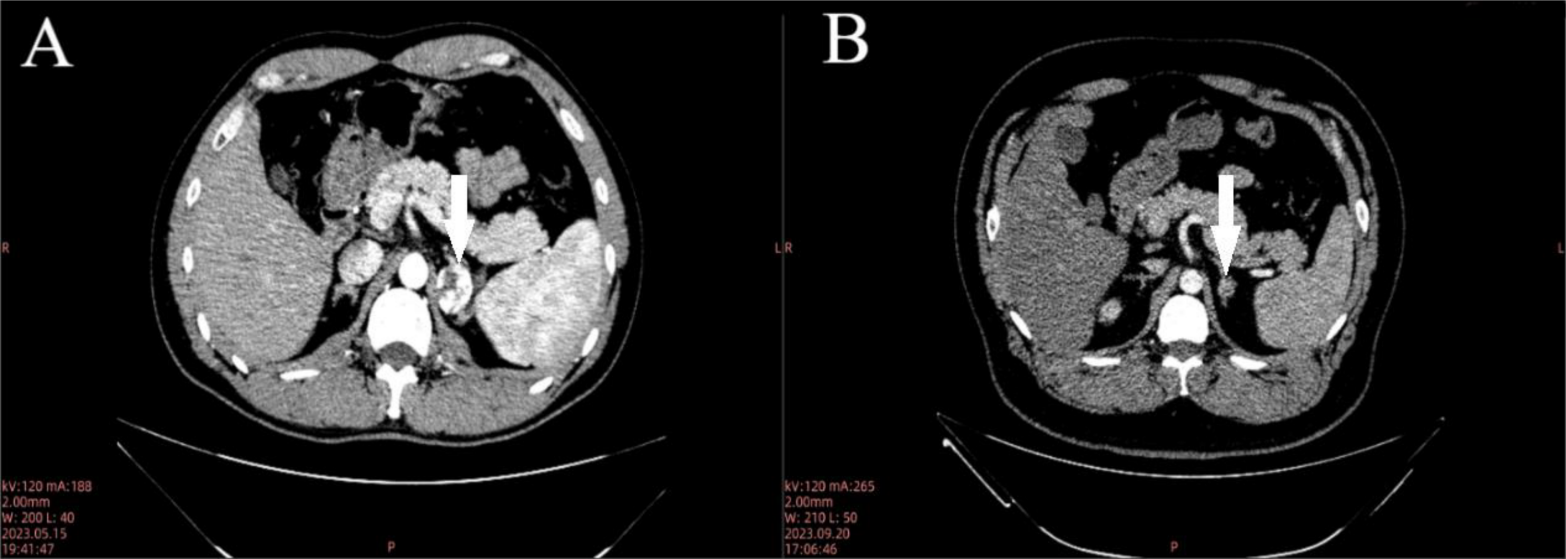
Enhanced CT images of Case 1 **(A)** and Case 2 **(B)**.

A 39-year-old male was admitted to the hospital three months after a computed tomography (CT) scan revealed a left adrenal mass. The patient did not exhibit symptoms such as hypertension or hypokalemia. The patient had undergone thyroidectomy for papillary thyroid carcinoma seven years prior and had been taking thyroid hormone replacement therapy since then. There was no family history of cancer. Physical examination revealed no palpable masses or tenderness in the bilateral renal areas. 24-hour urine catecholamine test, renin + angiotensin (supine and upright positions), plasma cortisol ACTH circadian rhythm testing, and thyroid function testing were all normal. CT scans showed an oval soft tissue density shadow in the medial branch of the left adrenal gland with a clear boundary and a low-density center, the mean CT value was 33 HU, measuring approximately 2.13×2.92 cm. (**Figure 1**) After enhancement, the edge showed significant enhancement, the mean CT value in the arterial phase is 102 HU. MRI scans revealed a round T1 slightly long, long T2 signal shadow in the left adrenal area, measuring approximately 2.6x2.3x2.4 cm, with moderate enhancement and no central enhancement. Radiologists considered a neurogenic tumor as a possibility. We engaged in an extensive dialogue with the patient, meticulously exploring the spectrum of potential diagnoses and the corresponding treatment avenues, including both the surgical removal of the mass and a conservative approach with vigilant monitoring. After this thoughtful and thorough discussion, the patient expressed valid concerns regarding the ambiguity surrounding the adrenal mass. Weighing the options carefully, the patient ultimately chose to pursue surgical resection as the most definitive course of action.

**Figure 2:**
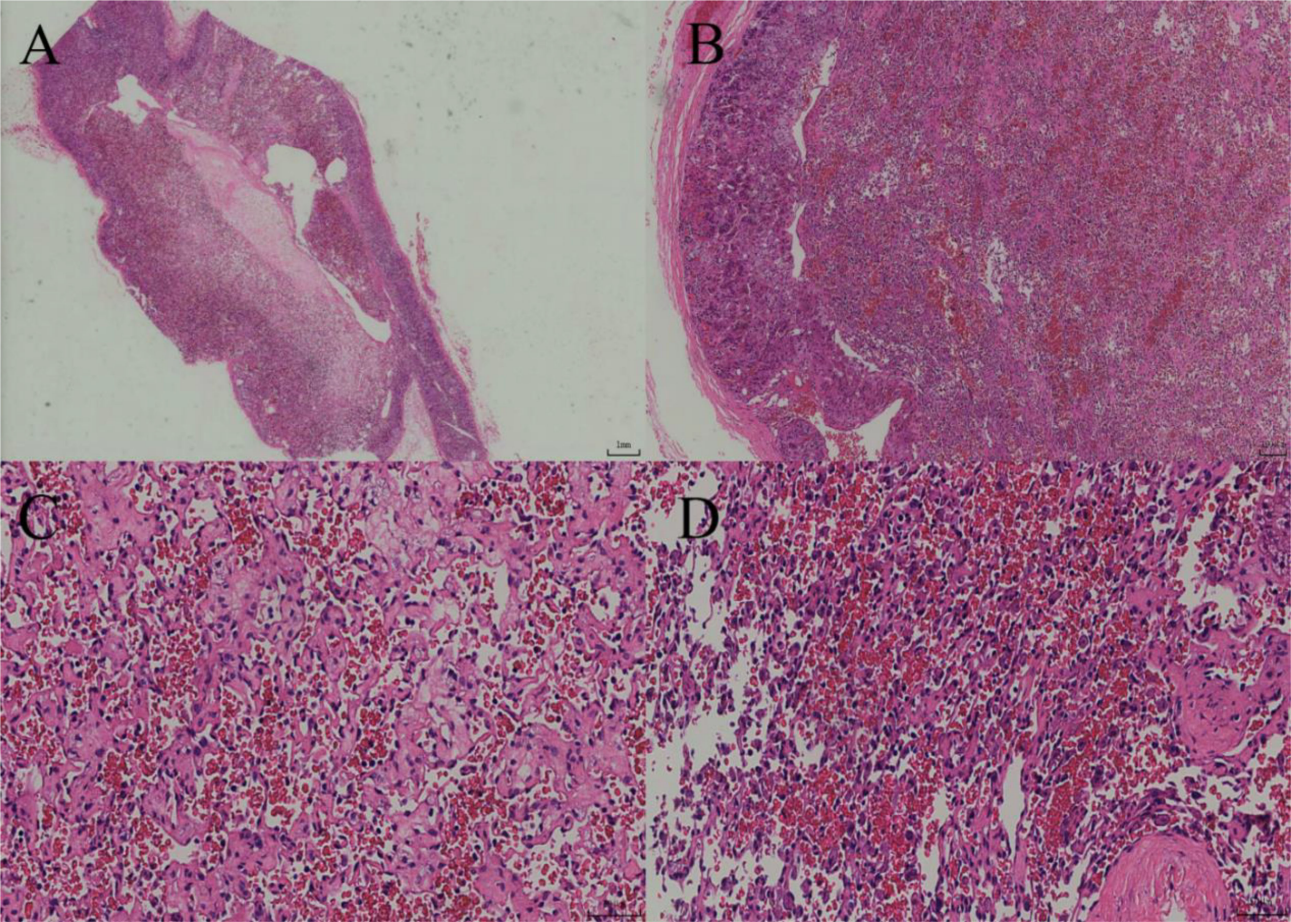
**(A)** Under low-power magnification, the tumor exhibits diffuse growth with a clear boundary and a complete capsule. **(B)** Under intermediate-power magnification, the tumor is located within the adrenal parenchyma, and luminal-like structures are visible between it and the normal adrenal tissue. **(C)** The tumor consists of irregular and variously sized capillary networks, which are interwoven and arranged in a crisscross pattern. **(D)** Under high-power magnification, the tumor is lined with a single layer of round to oval endothelial cells exhibiting a cobblestone-like appearance.

**Figure 3:**
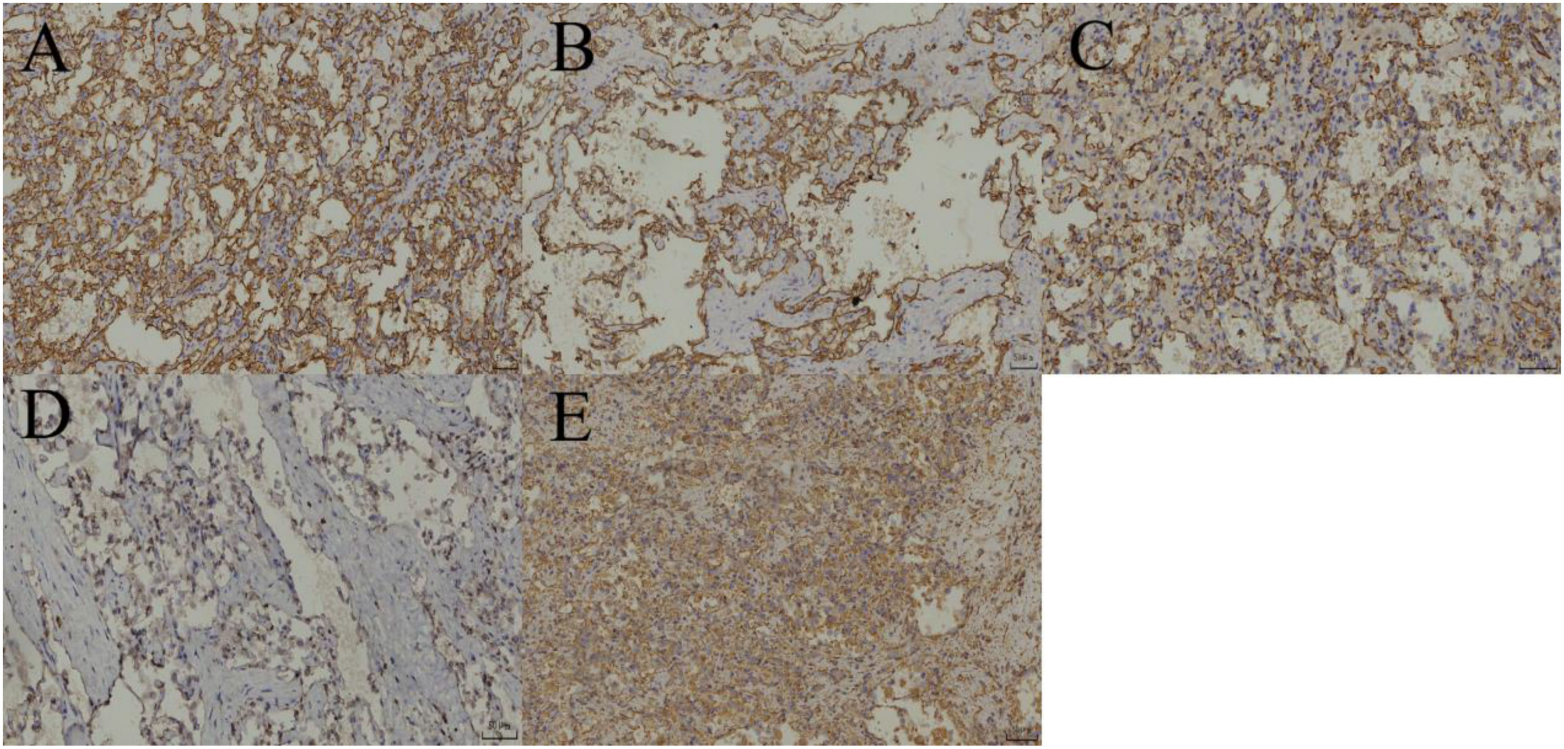
**(A–E)** CD31(+), CD34(+), F8(+), Fil-1(+), VIM(+).

After adequate preoperative preparation, laparoscopic partial adrenalectomy was performed. During the surgery, the Gerota’s fascia was opened longitudinally next to the psoas major muscle, and the upper pole of the kidney and the medial branch of the adrenal gland were separated using a combination of blunt and sharp dissection with an ultrasonic scalpel to expose the adrenal gland and the tumor. The tumor, measuring approximately 3 cm, was well-circumscribed from the surrounding tissue. After careful dissection, the tumor was completely resected and removed using a specimen bag. Grossly, the left adrenal mass was a gray-yellow to gray-brown soft tissue mass, measuring 4x2.5x2 cm. On cutting the largest surface, a gray-red, soft tumor measuring 2x1.5x1 cm was visible, well-circumscribed from the surrounding tissue. The pathological images and immunohistochemical stains are shown in **Figures 2**, **3**, respectively. The patient recovered well and was discharged on the third postoperative day. His treatment plan included follow-up visits to the urology clinic. CT imaging has not revealed any abnormalities in the left adrenal gland during 15 mouths follow-up.

## Case report 2

A 33-year-old male was admitted to the hospital one month after a CT scan incidentally revealed a left adrenal mass. The patient had a six-year history of hypertension and good blood pressure control with oral medication. The patient also had a two-year history of type 2 diabetes and normal blood glucose levels with regular subcutaneous injection of liraglutide. The patient had undergone thyroidectomy for thyroid tumor six years prior and had been taking thyroid hormone replacement therapy since then. There was no family history of cancer. Physical examination revealed no palpable masses or tenderness in the bilateral renal areas. Urinary catecholamine metabolite testing, renin + angiotensin (supine and upright positions), plasma cortisol ACTH circadian rhythm testing, and thyroid function testing were all normal. CT scans showed a nodule in the medial branch of the left adrenal gland, the mean CT value was 34 HU, with a longest diameter of approximately 1.4 cm, and significant heterogeneous enhancement after enhancement, the mean CT value in the arterial phase is 62 HU. (**Figure 1**) Radiologists considered pheochromocytoma as a possibility. After detailed discussion with the patient, surgical removal of the mass was decided, with the patient taking phenoxybenzamine orally for two weeks beforehand.

**Figure 4:**
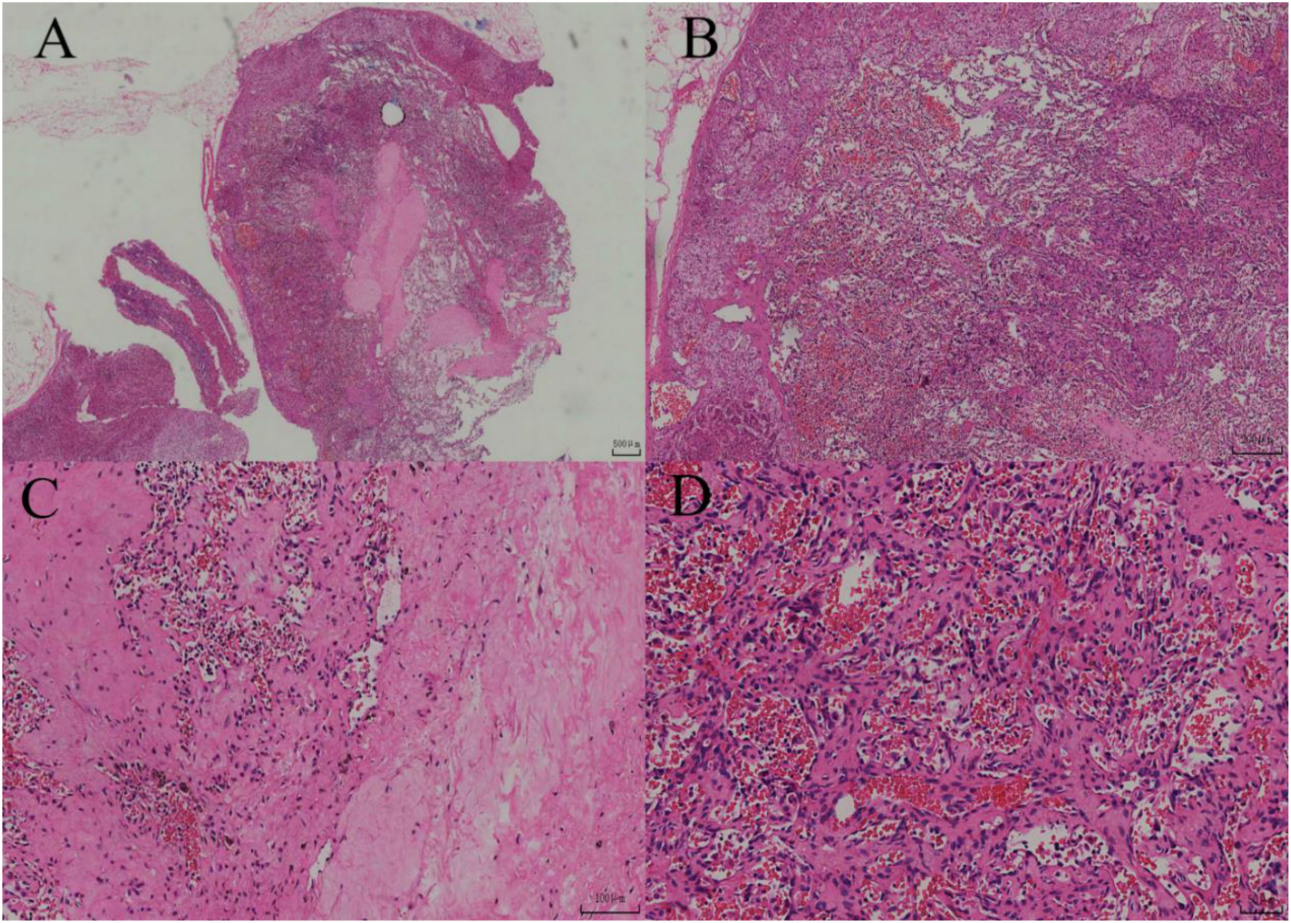
**(A)** Under low-power magnification, the lesion demonstrates diffuse distribution with a clear boundary and partially intact capsule. **(B)** Under intermediate-power magnification, the lesion is located within the adrenal cortex and exhibits a hemangiomatous appearance. **(C)** Vascular lumina with hyalinization and hemosiderin deposition are visible within the lesion area. **(D)** Under high-power magnification, the lesions are interwoven, lined with quasi-circular endothelial cells, and contain numerous red blood cells within the luminal spaces.

**Figure 5:**
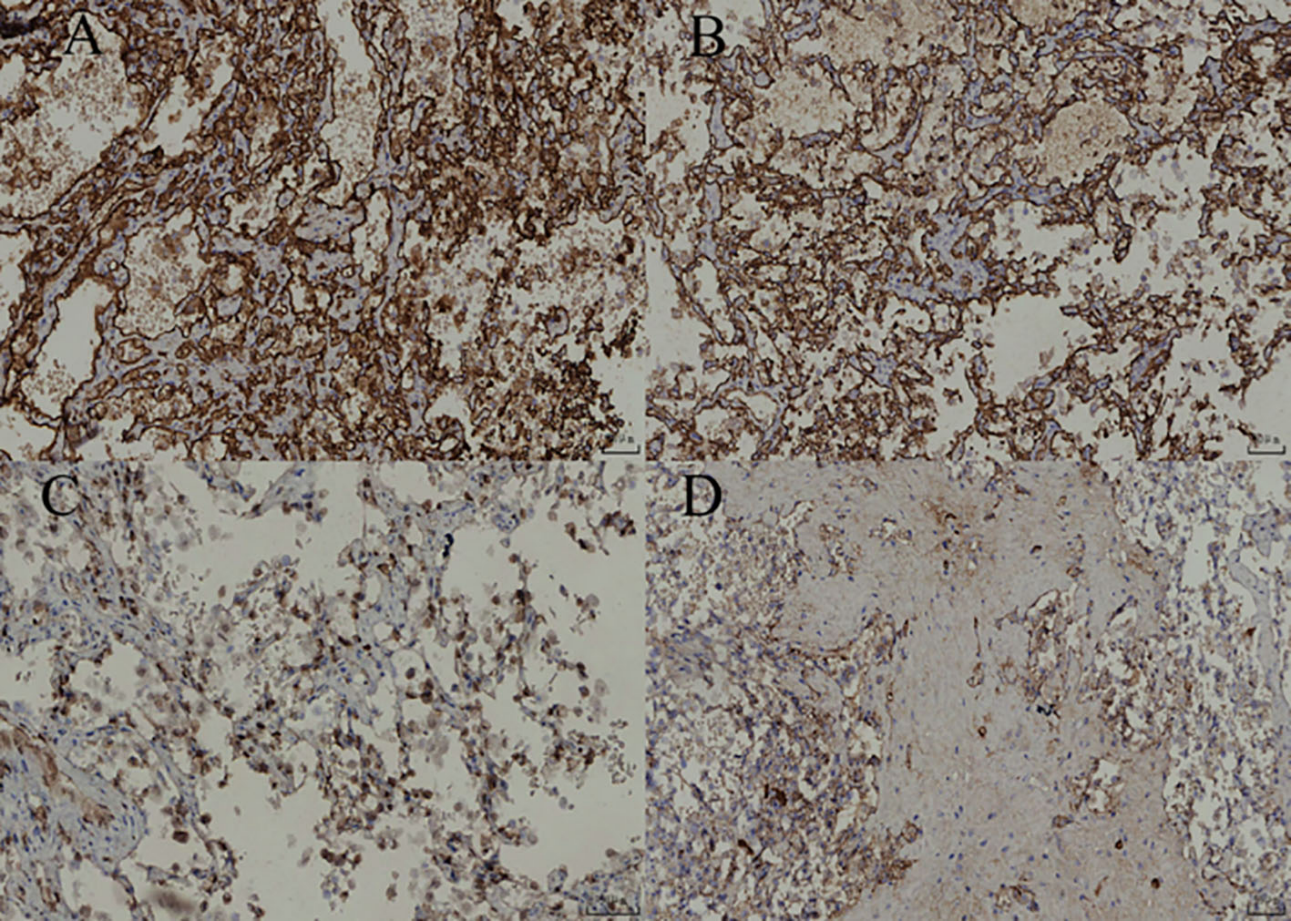
**(A–D)** CD31(+),CD34(+),ERG(+),F8(+).

During the surgery, the same procedure as in Case Report 1 was followed to expose, dissect, and resect the tumor. Grossly, the left adrenal mass was a gray-yellow to gray-brown soft tissue mass, measuring 2.5x2x1 cm. On cutting, the section was gray-yellow and soft. The pathological images and immunohistochemical stains are shown in **Figures 4**, **5**, respectively. Similarly, CT imaging has not revealed any abnormalities in the left adrenal gland during the 18 mouths follow-up period.

## Review of related literature

### Clinical characteristics

AH is a rare subtype of capillary hemangioma first reported and named by Montgomery and Epstein in 2009 as anastomosing hemangioma of the genitourinary tract, named for its characteristic irregular, interconnected, sinusoidal vascular lumina. In the 2016 WHO Classification of Tumors, AH was officially included as a unique subtype of renal hemangioma (4). AH is usually incidentally found during physical examination or other investigations and may rarely present with nonspecific symptoms such as hematuria and abdominal pain. The age of onset of AH ranges from 5 months (5) to 85 years, with a median age of 60 years, and it is slightly more common in males than in females. AH was initially found in the kidney and testis and has subsequently been reported in the adrenal gland, nasal cavity, liver, gastrointestinal tract, ovary, spermatic cord, para-aortic tissue, bone, and other locations. The kidney is the most commonly involved organ in AH, and nearly 40% of renal AH occurs during the follow-up of end-stage renal disease (ESRD) (6). Outside the kidney, soft tissue and bone, especially the paravertebral region, are the most common sites. AH of the adrenal gland is extremely rare, first reported by Michael (7). The two cases of adrenal AH reported in this study were both incidentally found during physical examinations, with no related clinical symptoms, and incorrect preoperative radiological diagnoses, highlighting the challenges in diagnosing this tumor.

### Imaging features

On CT scans, adrenal anastomosing hemangiomas typically appear as well-circumscribed, round to oval, soft tissue masses with slightly lower density. Previous reports of retroperitoneal AH have unenhanced CT values of approximately 15 HU to 37HU, consistent with our reported cases. Larger tumors may show heterogeneous enhancement after contrast administration due to the presence of fat or other components. On MRI images, anastomosing hemangiomas clearly display their vascular components, hemorrhage, thrombosis, and fat components, with characteristic T1WI low signal, T2WI high signal, DWI equal to high signal, and marked enhancement on arterial phase, as well as centripetal filling enhancement on venous phase (8, 9). On ultrasound images, anastomosing hemangiomas are often round or oval, with well-defined margins and heterogeneous internal echoes. Abundant blood flow signals are visible under CDFI, and the enhancement pattern is “slow in and slow out.” (10, 11) On PET-CT images, anastomosing hemangiomas typically show mild or low fluorodeoxyglucose (FDG) uptake, with a relatively low standardized uptake value (SUVmax). Additionally, some studies have reported that lesions may exhibit specific uptake of somatostatin analogs (8, 9). Despite these findings, the non-specificity of these imaging features makes accurate diagnosis through imaging examinations challenging.

### Pathological features

AH is generally well-circumscribed, often without a capsule, and has a gray or reddish-brown, meaty or spongy cut surface. The tumor size ranges from 1 to 8 cm, is usually solitary, but can also be multiple, with up to 15 lesions reported. At low magnification, the tumor margin is clear, with diffuse growth and occasionally lobulated growth. The periphery of AH is surrounded by veins, and the interior of the tumor contains thick-walled muscular blood vessels. At high magnification, irregular, capillary-sized vascular lumina are visible, showing a prominent interwoven pattern; they are lined by a single layer of round to oval endothelial cells, often with a hobnail appearance. The luminal spaces commonly contain erythrocyte extravasation and fibrinous thrombosis, accompanied by interstitial edema, fibrosis, and mucinous changes, with glassy globules visible inside and outside some cells. Cytologically, the tumor cells are mild and mostly without significant atypia, occasionally showing focal mild atypia or slightly enlarged nuclei. Cytoplasmic eosinophilic bodies are common in renal AH tumor cells. Most AH cases show focal degenerative changes, sclerosis, or extramedullary hematopoiesis, as well as mature adipose tissue. Tumor cells express endothelial markers: CD31, CD34, ERG, FLI-1, and FVIII-Ag-related antigen are diffusely positive, while D2-40, Calretinin, CK5/6, and broad-spectrum CK are not expressed (12).

### Molecular research

Recent studies have identified recurrent GNAQ and GNA14 mutations in adrenal anastomosing hemangiomas, further elucidating the pathogenesis of this tumor (13, 14). Mutations in GNAQ, GNA11, and GNA14 genes are the main molecular characteristics of anastomosing hemangiomas. These genes encode the α subunit of heterotrimeric G proteins, which participate in mediating signal transduction between G protein-coupled receptors and downstream targets. GNAQ gene mutations are the most common in anastomosing hemangiomas, with a mutation frequency of about 45% to 66%, mainly occurring at codon 209. This mutation is also common in uveal melanoma and blue nevus. GNA11 gene mutations are highly homologous to GNAQ, with a mutation frequency of about 19% in anastomosing hemangiomas, also mainly occurring at codon 209. These gene mutations lead to the disruption of GTPase activity, resulting in the continuous activation of the mitogen-activated protein (MAP) kinase pathway. This continuous activation state promotes cell proliferation, survival, and protein synthesis, providing an important molecular basis for the angiogenesis and proliferation of anastomosing hemangiomas (13–16). Although these GNA family gene mutations are not specific to anastomosing hemangiomas, their discovery provides molecular evidence for differentiating anastomosing hemangiomas from angiosarcomas, which frequently undergo mutations involving KDR, TP53, PIK3CA, and other oncogenes/tumor suppressor genes, and supports its diagnosis as a benign vascular tumor. Unfortunately, no relevant gene testing was performed in our cases.

## Differential diagnosis

1. **High-grade Angiosarcoma**: Although high-grade angiosarcoma can exhibit anastomosing and communicating blood vessels as well as primitive blood lumina, with positive CD31 and other vascular markers, it is distinguished by significant cellular atypia, active mitoses, obvious infiltration, and necrosis. Additionally, small thrombi, extramedullary hematopoiesis, and mature adipose tissue can provide important clues for differential diagnosis.
2. **Kaposi’s Sarcoma**: While Kaposi’s sarcoma may also present with eosinophilic bodies or erythrocyte extravasation in the tissue, it primarily affects the skin or lymph nodes of immunocompromised elderly patients. The tumor component is predominantly spindle-shaped cells with strong HHV-8 expression, differing from AH.
3. **Angiomyolipoma**: Angiomyolipoma contains varying numbers of smooth muscle cells expressing HMB45 and Melan-A. It lacks the abundant anastomosing sinusoid-like capillaries found in AH, with larger-caliber and thick-walled vessels instead.
4. **Angiomatoid Hyperplasia Secondary to Clear Cell Renal Cell Carcinoma**: This condition often occurs in patients with end-stage renal disease, and its CT manifestations are similar to AH, making it difficult to distinguish between them radiologically. When the tumor undergoes degenerative changes, the number of tumor cells decreases significantly, and low-grade nuclei tumor cells are embedded in the angiomatoid hyperplasia area, which can be easily misinterpreted as artifactually vacuolated lymphocytes. Extensive sampling and immunohistochemical examination are necessary to identify the concealed tumor cells.
5. **Adenomatoid Tumor with Thrombosis Organization/Anastomosing Features**: This group of tumors, first reported by Liau et al. in 2020, overlaps morphologically with AH and often has similar genetic abnormalities. However, it predominantly affects the skin and subcutaneous tissue and lacks the common eosinophilic cytoplasmic bodies and extramedullary hematopoiesis features of AH.

## Treatment and prognosis

The current treatment for AH primarily involves surgical resection. Long-term follow-up studies have demonstrated that AH is a benign or indolent vascular tumor, with no evidence of recurrence or metastasis when the tumor is completely resected (17). The two cases of adrenal AH reported in this study have not shown recurrence or metastasis since surgery. For this type of non-functional, asymptomatic adrenal incidentaloma, according to the European Society of Endocrinology guidelines: for newly discovered adrenal incidentalomas (AI), their imaging characteristics (attenuation value, size, uniformity) and functional status (endocrine activity) should be first evaluated by unenhanced CT. If CT shows an attenuation value ≤ 10HU and uniformity, regardless of size, it can be diagnosed as a benign adenoma without further examination; if the attenuation value is > 10HU, it needs to be managed according to risk stratification: low risk (11–20 HU and <4cm) supplemented with imaging (FDG-PET/CT, MRI, or CT contrast-enhanced examination) or re-examination after 12 months; high risk (>20HU, ≥4cm, or uneven) requires multidisciplinary team (MDT) discussion and consideration of surgical resection, with exclusion of metastasis before surgery; intermediate features (such as ≥4cm+11-20HU or <4cm+>20HU) require MDT combined with additional imaging (preferably FDG-PET/CT) for further evaluation (18). Emerging urine/plasma steroid metabolomics can assist in excluding malignancy, with a negative predictive value of 99.7% when combined with imaging (>20HU and >4cm) (19). Percutaneous puncture biopsy plays an important role in AH in adrenal external tissues (20), but multiple professional association guidelines do not recommend the use of biopsy in the examination of patients with adrenal incidentalomas unless there is a history of adrenal external malignant tumors and suspected metastatic disease is suspected (18, 21, 22). Both cases reported here had a history of papillary thyroid carcinoma; however, given the excellent prognosis of this tumor and its predilection for regional lymph node metastasis, the adrenal tumors were not considered metastatic lesions. Due to the lack of accurate preoperative diagnosis, nearly 90% of AH cases are treated as malignant tumors. Therefore, it is crucial for surgeons to avoid unnecessary overtreatment, such as radical resection, extensive lymph node dissection, and unnecessary preoperative/postoperative chemotherapy or radiotherapy. Enhancing the understanding and preoperative diagnosis of this disease is significant for preventing overtreatment.

In conclusion, AH is a rare and poorly understood benign vascular tumor, particularly when located in the adrenal gland. It is crucial for clinicians and pathologists to recognize its unique features to facilitate accurate diagnosis and appropriate treatment, thereby avoiding overtreatment and ensuring optimal patient outcomes.

## Data Availability

The original contributions presented in the study are included in the article/supplementary material. Further inquiries can be directed to the corresponding author/s.
